# The Role of Contextual Factors in Shaping Urban Older Adults’ Intention of Institutional Care in China: A Mixed-Methods Study

**DOI:** 10.3390/ijerph20064731

**Published:** 2023-03-08

**Authors:** Yuekang Li, Jinbao Zhang, Hao Luo, Xiaomei Pei, Tao Wu, Jun Jing

**Affiliations:** 1 Brown School, Washington University in St. Louis, Saint Louis, MA 63130, USA; 2 Department of Social Work and Social Administration, The University of Hong Kong, Hong Kong 999077, China; 3 Department of Sociology, Tsinghua University, Beijing 100084, China; 4 Gallup China, Beijing 100022, China

**Keywords:** residential and care intentions, institutional care, living arrangement, aging-friendly community, mixed-methods

## Abstract

Background: This mixed-methods study explores older people’s intention of institutional care and its contributing contextual factors, and the meaning given to their intention by older adults in the transitioning Chinese society. Methods: Guided by the extended Anderson model and frameworks of the ecological theory of aging, survey data collected from 1937 Chinese older adults were used. Transcripts from six focus group interviews were analyzed to incorporate the voices of the participants. Results: Community environment and services, health services, financial services, and regional service organizations were related to the institutional care intention of older people. The qualitative analysis showed that the reported conflicting feelings about institutional care was driven by the lack of supporting resources and age-friendly environment. The findings of this study suggested that the reported intention of Chinese older adults for institutional care may not be an ideal choice but a compromise or, in some cases, a forced choice. Conclusions: Rather than treating the stated institutional intention as a simple expression of older Chinese people’s likes or desires, the intention of institutional care should be understood within a framework that fully considers the influence of psycho-social factors and contextual organizations.

## 1. Introduction

The last few decades have witnessed a paradigm shift from institutional care toward home- and community-based services in Western countries. It has been widely documented that most older people want to stay in their familiar physical and social environment as they grow older [1]. Aging in a self-preferred arrangement is beneficial for older people as it is associated with better physical and mental health, life satisfaction, self-esteem, and even reduced mortality [2]. Enabling people to remain in their homes and communities for as long as possible also avoids or delays the high cost of institutional care and is therefore favored by policymakers, health providers, and older people [3].

Compared with older adults in Western societies, older adults in China are suspected to be more likely to prefer aging in their own homes and community. This is because the care of older people in China has traditionally been confined to the familial sphere, long enshrined by the norm of filial piety [4]. Institutionalized older people have often been stigmatized because institutional care was only provided to welfare recipients with no families, no income, and no ability to work [5]. It was not until recently that the formal services for the older population increased to meet the escalating demands as the ability of Chinese families to take care of their older members was eroded by demographic shifts and socio-economic development [6]. In response to the rapidly aging population, the Chinese government issued a blueprint for the emerging aging services and support networks: home-based care as the foundation, community-based services as backing, and institutional care as a complement [7]. According to central planning, the proportion of institutionalized older people should be under 4%. However, recent studies have suggested that the proportion of older Chinese adults who reported an intention of institutional care has increased substantially, especially among city dwellers. Based on a national survey, Chou (2010) [4] reported that 20% of older people in cities intended to live in a residential care facility, in contrast to only 5% in 2003 [8]. A more recent study reported an even higher percentage, over 30%, using data from the 2014 China Longitudinal Aging Social Survey (CLASS).

Many factors may have contributed to the institutional care intention among older Chinese adults. Individual and family characteristics are key influences on an older Chinese person’s choice [4]. For example, Chinese older adults’ propensity to use institutional care is found to be consistently associated with their number of children and their relationship with their adult children [9]. The findings regarding health and functions are inconsistent, with several studies reporting functional impairments and health conditions associated with greater willingness to move into intuitional care homes, whereas others found these health-related factors were not significant determinants of preference [4,7]. Furthermore, psycho-social factors, such as filial piety belief and attitude toward institutions, play a role. Some researchers have proposed that the rapid social changes are altering the cultural norms regarding traditional family arrangements and causing older Chinese adults to favor institutional care as a source of independence and peer support [10], while others have reported that the negative connotations of institutionalization in China are still dominant and that older people are reluctant to live in residential care facilities [4,11].

What has largely been overlooked in previous research is the role of contextual factors, such as community environment and welfare organizations, in shaping older Chinese people’s residential and care intentions. In his ecological theory of aging [12,13], suggested that a decision to relocate can result from a mismatch between the pressure of the contextual environment and the competency levels of the individual. A supportive community environment may promote older people’s intention and ability to remain at home. A growing body of research has documented the effects of community services on the intention of institutional care. For example, having health facilities, community services, and senior centers within reach has been related to less institutionalization anticipation [11]. However, although the general policy guidance sets home- and community-based services (HCBSs) as the preferred mode of service delivery in China, the actual policy implementation has incentivized the development of institutional care by setting targets for bed supplies [5]. From 2008 to 2018, the total number of residential care beds more than tripled, in contrast to HCBSs, which remain underdeveloped, fragmented, and lacking coordination [14]. Older Chinese people may consequently adapt their intention to fit their only available option—institutional care. However, whether the increasing intention of institutional care reflects the genuine tendency of older Chinese people or is a need related to the absence of home- and community-based support is unclear.

This question arises because the past literature has reported quantitative investigations focusing on the factors associated with the reported residential and care intentions but failed to incorporate participants’ voices to describe the meaning behind the stated intentions. Because the institutional care intention of older adults may not be a simple expression of their likes or desires and may be influenced by psycho-social factors and contextual organizations [4,15], there is a need to gain a more comprehensive understanding by synthesizing both quantitative and qualitative data.

This mixed-methods study aims to investigate the intention of institutional care among older Chinese adults and the role of contextual factors in shaping this intention. More specifically, we aimed to investigate: (1) whether the intention of institutional care is associated with contextual factors, such as the community environment, community services, and regional service organizations; and (2) what Chinese older adults’ perceptions of institutional care and experiences of aging in the current environment are and how these perceptions and experiences influence their care intention.

### Conceptual Framework

The conceptual framework for this study was adapted from the extended Anderson model and frameworks of the ecological theory of aging. The Anderson model [16] and its extensions [17] postulate that service utilization is determined by predisposing factors, such as age, gender, and education; need factors, such as perceived and functional ability and general health condition; enabling factors, such as the availability of support and financial resources; and psycho-social factors, such as attitudes and beliefs regarding service use and knowledge of and familiarity with services.

Based on the ecological theory of aging [13], we identified two layers of contextual factors. The first layer concerns the way in which the immediate environment is associated with older people’s residential and care intentions. The second layer of contextual factors relates to the availability, priority, and structure of services at the local level, which are beyond personal characteristics [13]. The higher-level contextual factors may influence individuals by changing cultural norms and beliefs or altering aspects of the more immediate environment. In this paper, the second layer of contextual factors is operationalized as the regional differences and variations in social care service provision.

## 2. Materials and Methods

### 2.1. Study Design

We employed a convergent mixed-methods approach to understand the association between complex contextual factors and Chinese older adults’ intentions for institutional care. Following this approach, we used survey data to quantify the relationship between individual- and contextual-level factors and the intention of institutional care. Focus group interviews were conducted to gain a more comprehensive and in-depth understanding of how older Chinese people’s social context and actual lived experience related to the stated institutional care intention. The convergent mixed-methods approach encouraged a dialogue between quantitative and qualitative methods [18] and the integration of mixed methods occurred at different points. During the research design stage, both the qualitative and quantitative questions were guided by the same conceptual framework and were interrelated. For example, both focused on to what extent the environmental resources met the needs of older adults. During the data analysis phase, we first analyzed the quantitative data while reflecting on the qualitative narratives and looked to the qualitative narratives to inform our understanding of the quantitative findings. Then, based on the primary analysis, the final stage of interpretation reflected on the ‘‘whole’’ that was produced from and transcended the individual ‘‘parts’’ [19].

### 2.2. Data Source

The participants selected for the present study were part of the Gallup-Tsinghua Elderly Care Study (CESS) conducted between March and November 2016, including both the quantitative and qualitative parts. The CESS aimed to investigate older Chinese people’s demands for health and social care services to provide policy recommendations. This research has been approved by the Institutional Review Board at the Research Centre for Medical Sociology, Tsinghua University(ID:201516).

### 2.3. Quantitative Research

#### 2.3.1. Quantitative Data Collection

The quantitative data were collected through a telephone survey. Participants were from 16 cities in China, consisting of four metropolitan cities (Beijing, Shanghai, Guangzhou, and Shenzhen), eight major cities (Qingdao, Fuzhou, Dalian, Xi’an, Chengdu, Chongqing, Wuhan, and Lanzhou), and four medium-sized cities (Fuyang, Loudi, Yangquan, and Jiaxing). These cities were purposely chosen to cover various levels of economic development and a wide range of geographical locations. The interviewers initially reached 68,000 people by randomly dialing 270,000 household phone numbers in 16 cities. Respondents were included if they were aged 60 years or above, were cognitively intact, had no hearing problems, and consented to participate in the study. No personal identifiers were obtained during this survey. A total of 2018 participants completed the 20-min telephone interview. Because this study only focused on city-dwelling older adults, rural older adults were excluded, and the final sample consisted of 1937 respondents. We also conducted power analysis to examine to what extent we have confidence in the model results presented. Results of power analysis suggested achievement of a power of 0.8; a minimum sample size is 491 cases. Because the sample size of this study is much more than 66 older adults (*n* = 1937), it is safe to assume this study has adequate power.

#### 2.3.2. Measures of the Quantitative Study

##### Dependent Variable

The dependent variable was institutional care placement intention. The participants were asked, “where do you want to stay as you age?”, and there were five possible responses: (1) staying in their own home; (2) moving to a children’s home; (3) depending on community daycare centers; (4) living in institutions, and (5) others. We collapsed this variable into a dichotomous variable to indicate whether older persons preferred institutional placement.

##### Independent Variables

The independent variables considered for the multivariate analysis included predisposing, psycho-social, enabling, and need factors.

##### Predisposing Factors

Age was categorized by five-year age groups: 60–64, 65–69, 70–74, 75–79, 80–84, and 85 or over. Education attainment was a five-scale categorical variable: below primary school, primary school, secondary school, high school, and college or above.

##### Psycho-Social Factors

Respondents’ attitude toward public and private institutions was elicited through the question “What is your overall impression of public/private institutions for older people?” and was responded to with a choice of four answers: poor, average, good, and not sure. Knowledge of public and private institutions was obtained through the question “Do you know about public/private nursing homes?” and was answered with three options: do not know, partially know, and know.

##### Enabling Factors

- *Individual Resources*

The enabling factors comprised individual and environmental resources that served to satisfy older people’s needs and were measured at the individual and regional levels. For the individual factors, marital status indicated whether spousal assistance was available. Household size was also included to denote the availability of family support. According to the participants’ self-assessment, income was categorized into four types: maintaining life with great difficulty, a little short of money, successfully maintaining life, and living a comfortable life. Social support indicated whether participants could receive assistance from friends and neighbors in the event of an emergency. The variables used in this study are consistent with other studies using the Anderson model [17].

- *Community Resources*

We adopted the age-friendly community initiatives (AFCIs), a community-level strategy guided by the ecological perspective, to conceptualize and operationalize the community-level environment and investigate whether communities can meet the needs of older adults and allow them to remain involved in community life [20,21]. AFCIs’ characteristics include proximally located goods, services, and amenities; the availability of transportation options; safe and accessible neighborhoods and housing; access to sources of social support; and opportunities to engage in meaningful activities [21]. In this study, the participants reported whether they were satisfied with five age-friendly characteristics, including public transportation, housing, medical services, community services and environment, and fitness and recreation facilities. The answers were binary: satisfied and dissatisfied.

- *Welfare Organization*

The regional-level welfare organization data were collected from the 2016 official yearbooks: The Civil Affairs Statistical Yearbook, the Population & Employment Statistics Yearbook, and the Health and Family Planning Statistics Yearbook. We focus on the regional-level provision of two types of services: HCBSs and institutional care. The supply of HCBSs was measured using the number of daycare center beds, activity centers, and community health care beds per thousand older adults at the provincial level. Institutional care provision was gauged through the number of institutional beds per thousand older adults at the provincial level.

##### Need Factors

Needs were measured at the individual and the regional level. Individual needs were measured through self-reported health status and needs for assisted help in daily living (ADL). The self-reported health status was classified into two categories: healthy and unhealthy. Participants who reported needing assistance with daily activities, such as eating, bathing, dressing, and toileting, were considered to have ADL needs. The provincial population needs were calculated as the number of older people aged 65 or above divided by the number of people aged 15 to 64. All the metrics at the provincial level were centered for ease of interpretation.

##### Quantitative Analysis

Chi-square tests and t-tests were performed to assess the differences between respondents who preferred institutional placement and those who did not. Multilevel logistic regression analysis was conducted as regional-level data were used. In addition, the value of variance inflation factors (VIFs) showed that there was no risk of multicollinearity (VIF < 10 and mean VIF = 2.26). Multiple imputations with chained equations were used to impute missing values in control variables and the depressive symptoms variable (the largest percentage of imputed value: 5.27%). All the analyses were conducted using Stata Version 15. Statistical significance was set at the 5% level.

### 2.4. Qualitative Research

#### 2.4.1. Qualitative Data Collection

Following receipt of ethics approval from the relevant institutional review board, the qualitative phase included 52 older adults (30 females and 22 males) who volunteered in response to recruitment made by local community agencies (such as senior centers and neighborhood committees) of five neighborhoods in Beijing, Shanghai, and Guangzhou. The average age was 73.11 years old. The five selected neighborhoods all had high proportions of the older population but differed in terms of their socio-economic status, geographic settings, quality of service infrastructure, and cultural context. Eligibility criteria included 60 years and above, demonstrated cognitive capacity to participate, having no hearing problem, and consenting to participate in the study. The staff of local community agencies worked with researchers to ensure eligible participants were cognitively and psychologically capable of completing the interview. These 52 participants formed six focus groups. Each group contained 8 to 10 persons and was formed by residents living in the same neighborhood. It was assured that the participants in each focus group were diverse in many respects, such as age groups, living arrangements, family relationships, health conditions, and working and welfare status.

One researcher served as the focus group moderator, while two others observed the group discussions. They asked open-ended questions about various possibilities and options for aged care services and facilities that were available, as well as the participants’ likelihood of using them, their abilities, and their options for paying for these services. The focus group questions were guided by the Anderson model and the framework of the ecological theory of aging, and also referred to previous relevant studies [22,23,24]. Some example research questions are the following: What difficulties and inconveniences have you encountered in your daily life? How would you like to be cared for in your later life and why? What is your opinion and experience about institutional care and other aging-related services? What is the most important factor to consider when choosing aging-related services? What unmet needs do you have, and what services do you expect? These focus group interviews lasted about one hour. All interviews were carried out in Chinese, recorded, transcribed, approved by the participants, and then analyzed line by line. At the beginning of the interview session, all participants were informed about the study and signed a consent form to participate in the study. All the interviews were anonymized at the time of initial transcription and pseudonyms or replacements were used to make sure no personal identifiers can be obtained by researchers.

#### 2.4.2. Qualitative Data Analysis

The qualitative study employed an inductive thematic analysis of the narrative data. Coding was carried out by carefully reading the transcript line by line, reflecting on the meaning of the text, and themes that emerged through constant interview comparisons. Because this study was guided by theoretical frameworks, the coding proceeded using a “hybrid” approach integrating deductive codes derived from our theoretical framework, with data-driven inductive codes. This approach was essential for addressing the research question, as it allowed us to explore how contextual factors influence older people’s intention of institutional care while allowing codes to be data-driven. We sought to enhance this study’s rigor by frequent discussions as a team throughout all phases of the data analysis process. Researchers brought perspectives from social work, gerontology, sociology, and anthropology to the data analysis process, thereby enhancing theoretical sensitivity [25].

## 3. Results

### 3.1. Quantitative Results

Table 1 shows participants’ characteristics by institutional care intention. Most participants were male (58.75%), aged between 60 and 74 (65.30%), and had less than a college education (73.24%). Participants reported poor health status and ADL needs, accounting for 30.10% and 5.73% of the total sample, respectively. Participants who reported an intention of institutional care were more likely to be more educated, have a positive attitude toward public and private institutions, have more knowledge about private institutions, live in a small household, and be less satisfied with AFCI factors.

Table 2 shows the characteristics of welfare organizations based on institutional care intention. Participants living in regions with fewer daycare center beds tended to express an institutional care intention.

Table 3 presents the results of the logistic regression. Male participants were more likely to prefer institutional care than their female counterparts (OR = 1.54, 95% CI 1.14–2.06). Compared with those having education below primary school, participants with higher than primary education attainment were more likely to choose institutional care. Older people with positive attitudes toward public institutions and better knowledge of private institutions were prone to report an institutional care intention.

Regarding the AFCIs, older adults perceiving that better health services (OR = 0.65, 95% CI 0.48–0.87) and better community services and environment (OR = 0.71, 95% CI 0.52–0.98) were less likely to elicit an intention of institutional care. Regarding regional factors, the provision of different types of services influenced participants’ intention of institutions. A greater supply of institutional care was related to a greater likelihood of indicating an institutional intention (OR = 1.02, 95% CI 1.00–1.05). In contrast, more available daycare centers were related to a lower tendency to report an intention of institutional care (OR = 0.56, 95% CI 0.37–0.85).

### 3.2. Qualitative Themes

#### 3.2.1. Conflicted Feelings about Institutional Care

In the focus group narratives, a majority of participants showed an intention to choose institutional care. For example, a few Guangzhou participants expressed that they would, as older people, enter institutions sooner or later. Some participants had started to look for an institution for themselves, although none had any long-term care (LTC) needs at the time. There was a distinction between public and private institutions. The participants confirmed that public institutions provide cheaper and trustworthy services but that “it is difficult to get in; people have to wait three to five years for a vacancy.” Therefore, the participant narratives mostly revolved around private facilities.

Despite the strong intention of institutional care, the participants did not show a positive attitude toward LTC facilities; instead, concerns and distrust were brought into the discussion. Most of the participant narratives mentioned the low quality of care. For example, a Shanghai participant mentioned:

I heard that older people in nursing homes have ulcers because the workers did not care for them carefully. You have to pay extra money to find a good care worker; otherwise, you suffer.

The participant narratives also revealed that privately-owned institutional care is beyond the affordability of regular families. In China, most facilities are under the administration of the civil affairs offices, and it is difficult for these facilities to obtain certification for delivering medical services. This means that most of the facilities’ services cannot be covered by medical insurance. This situation also aroused concerns about the extent to which the facilities can provide adequate medical services. It was reported that some facilities, due to limited nursing capability, refused to accept dependent older adults, although they need this service the most.

#### 3.2.2. Lack of Family Resources

Participants plan to live in a nursing home despite holding doubts because they believed that the younger generation, who served as the traditional sources of caregiving, could no longer fulfil the caregiving role. As narrated by a participant living in Guangzhou:

If I become dependent, I will go to the nursing home because if you stay at home and hire a domestic helper, it burdens your child. I don’t want to trouble my child because they have their family, right?

Similar narratives were repeated in other groups. The “one-child” policy was often mentioned simultaneously with care intentions. For example, a female participant living in Beijing responded to the question about her future care plan:

We all have only one child, just one. We cannot rely on children because they have work to do. If you rely on them, they may lose their job. This is not good.

The above narrative illustrates that, although the participants recognized the family caregiving tradition, they also believed that the younger generation could no longer fulfil the caregiving role.

#### 3.2.3. Lack of Community Resources

Since the changing family structure has eroded the availability of family care, participants expected the community could support them in later life. However, though none of the participants were experiencing functional limitations at the time, many still stated that older adults face “endless” inconveniences in daily life. The participants expressed difficulties accessing a wide range of social and health services, such as transportation, financial, and medical services. A couple of participants believed that older adults had been increasingly excluded from basic services as everything has become digital. For example, participants living in Beijing described the difficulty of seeing a doctor:

It isn’t easy to see a doctor. Someone told me that I should make an appointment online. We can’t even deal with mobile phones; how can we know the Internet? I think we are all illiterate now.

Unable to access the services that are increasingly being delivered through the Internet, older adults have to put in a lot of extra effort to have their needs met:

I am almost 80 years old. The hospital opens at 7 a.m. I waited there at 6:30 but still failed to make an appointment. I begged the doctor to add me to the line. I just wanted to check my medicine dose. Luckily, the doctor agreed. So I waited in the hospital until 12:30 p.m. The doctor took a look at my record and told me to keep the same dose: just one sentence. I had waited for six hours.

It was widely agreed among the participants that comprehensive community health centers are urgently needed because, currently, older adults with health issues cannot stay in the community. There are hardly any community-based LTC services—only one participant had some experience with adult daycare. According to her, there was a great demand for such services. However, older adults in her community stopped attending the daycare center because only lunch was provided, and that was not enough to support older people who could not cook.

The inaccessibility and unavailability of community-based services seem to exacerbate participants’ sense of insecurity and anxiety about growing old. A common fear among the participants, regardless of their health conditions, was shown by the following narrative:

We can go nowhere to seek help for sudden illnesses, and there is no supporting system in the community. I am anxious; I may die alone.

The participant narratives raised the question again of whether the strong intention to opt for institutional care reflects the true preference of Chinese older adults or the anxiety about growing old due to insufficient neighborhood support. There was a repeated pattern in the interviews whereby, while agreeing that everyone will end up in institutions in the near future, the participants also stated that they would rather live in the community if they could. For example, a Beijing participant stated:

We have been looking for institutions but want to stay at home. We want to stay in a small group of familiar people. The question is how community health services can keep up with our needs.

Another Shanghai participant echoed her opinion:

I hope to stay at home when I get older, and the home service is the most preferred. But it is getting harder to find a domestic helper, and the price is so high; this is our biggest headache. You don’t necessarily want the government to serve us older people more; I mean, building many nursing homes, but that is not the case.

Here the participant mistakenly identified aging service provision with building nursing homes and did not view HCBSs as an option. He also identified HCBSs with private domestic helpers and regarded this need as his own responsibility. This may reflect the influence of an institutionally biased welfare organization on individuals, older adults, and their families, who are misled into believing that institutional care is the only option. If family caregiving is unavailable, most participants have to confront a dilemma between hiring a domestic helper and moving to an institution for the rest of their life, both of which require out-of-pocket payment. As the Guangzhou participant mentioned earlier, he still wanted to stay in a nursing home, although he did not like it, “Because if you stay at home and hire a domestic helper, it will burden your child, right?”

However, we also found an emerging expectation for social HCBSs among older adults. For example, a participant living in Beijing suggested:

I hope an aging service station will be set up in our community. This service station can take care of all aspects of the resident’s daily life.

Other participants also proposed that, considering the lack of funding and personnel in communities, older people living in the same neighborhood may help each other. However, this can be made possible only with community-level effort.

## 4. Discussion

This mixed-methods study set out to understand Chinese older adults’ intention of institutional care in the complex social, cultural, and policy contexts. Guided by the Andersen model and the ecological perspective, the quantitative phase of this study indicated that using a relatively representative sample of Chinese urban older adults, contextual factors such as the community environment, services, and regional service organizations were related to institutional care intention. The findings of the qualitative phase were consistent with the quantitative findings and further illustrated that people have conflicting feelings about institutional care, meaning they have negative feelings about it but also prefer it, which was driven by the lack of supporting resources and age-friendly environment in the cities. The findings of this present mixed-methods study suggested that the reported intention of Chinese older adults for institutional care may not be an ideal choice but a compromise or, in some cases, a forced choice.

Contrary to the Andersen model’s hypothesis that service-seeking behavior is fundamentally dependent on needs, none of the need factors, including self-reported health, ADL difficulties, and the predisposing age factor, which is positively associated with ADL needs, were related to the intention of institutional care. Focus group participants who were functionally intact also showed great intentions and concerns about institutional care. This finding concurs with other Chinese studies [4,26], but seems to be a unique phenomenon in China as need factors were constantly found to be associated with the intention of institutional care in studies conducted in other countries [27]. One possible explanation might be that the utilization of institutional care in China is still based on the extent of the service user’s ability to pay the market fees rather than a need assessment based on functional impairment [14]. Consequently, frail older persons with severe physical impairments are reluctant to live in institutional settings where the quality of care cannot live up to their expectations.

On the other hand, this study explored the residential intention from the stated preference approach, which is focused on the ideal choices that individuals and households would make to face hypothetical situations (Fernández-Carro 2016). The choices do not imply an authentic decision but are opinions about expectations and goals. Findings from other countries have shown that younger older adults have different perceptions of the potential risk of being placed in a nursing home than older cohorts because the placement is not yet on their immediate time horizon [27]. However, we observed in our data that institutional care placement is on the radar of individuals of all ages and health ranges.

A possible reason might be that the family has long been the primary and culturally favored source of caregiving [5]. It is the first time in history that demographic shifts and socio-economic changes have increasingly eroded the capacity of Chinese families for care. In this circumstance, older Chinese parents increasingly fear becoming a burden on the younger generation. The growing interest in formal assistance reflects older people’s attempts to solve the new challenge on their own and a shifting attitude toward the very concept of filial piety [28]. Older people’s willingness to live in institutional care may imply a compromise between their desire and the actual possibilities [4]. Therefore, the institutional care intention of older adults in a transitioning familistic society should be understood within a framework that fully considers the influence of psycho-social factors and contextual organizations [15] rather than treating the stated intention as a simple expression of a person’s likes or desires.

Interestingly, the participants’ knowledge of private institutions affected their choices, but their attitudes did not. The opposite was true for public institutions. Our focus group data help us to understand this phenomenon by revealing that, while older adults generally trust the government’s LTC services, they distrust private institutions and report general concerns about the quality of the services, their personnel, and their affordability. Older people may deem private homes to be the option of last resort and their personal dislikes not to be necessary.

Attitudes and knowledge are crucial to achieving the enhanced autonomy of older people in their own decision-making [15]. However, their attitudes must also be considered within the contextual organizations. Our findings show that the ability of older people to remain in their own homes is restricted by the unsupportive environment and insufficient service provision, especially community-level health services. The mismatch between low literacy, undesirable views, and stated institutional intention may be due to the absence of preferred alternatives. Ideally, AFCIs can help prevent or delay the onset of illness and disability, reduce the stress on caregivers, and help avoid premature and unnecessary institutional care [3]. However, the Chinese aging service system is dominated by institutional care and lags in developing HCBSs and an age-friendly environment [6]. The fundamental problem with such a system is that institutional care targets a small proportion of older adults who already have illnesses and disabilities. Over-generalized provision of institutional care may reinforce the stereotype that all older adults should depend on others, in contrast to AFCIs that aim to enhance older adults’ capacity to function optimally in their own homes and communities and prevent problems from occurring in the first place [20]. Such policy runs the risk of creating an aging service system that forms dependence and vulnerability among older people. The influence of the policy priority on older individuals’ choices may be more significant than expected. Illustrated by this study, although the number of daycare centers was minimal compared with the availability of institutional care at the provincial level, older people living in areas where slightly more resources were allocated to daycare centers still reported a lower intention to choose institutional care.

### Limitations

The present study has several limitations. First, the participants were limited to 16 relatively larger cities. Thus, we should be cautious about generalizing our results beyond the study sample, especially to older adults from rural and small towns. Although the participants were recruited by randomly dialing phone numbers, selection bias may still exist as only older adults reached by landline were involved. Second, since the telephone survey was restricted to 20 min, the institutional care intention was measured by a single question. Other variables, such as AFCIs’ characteristics and psychosocial factors, were measured using selected questions rather than a holistic assessment tool. Future research should consider including validated scales to increase the accuracy and validity of the data. Third, though the focus group may encourage discussions among participants, it may also overestimate the intention for and anxiety related to institutional care as the tendency to build upon the idea may center around a few individuals’ contributions in a group discussion. Therefore, the reported intention in the focus group was higher than that of the survey. Future studies may consider an in-depth individual interview as the method to collect qualitative data.

## 5. Conclusions

Despite the limitations, our study is the first to have applied a mixed-methods design and has been guided by an integrated model to examine older Chinese people’s intention of institutional care. The results from this study have substantive theoretical and policy implications for residential and care arrangements for older adults in China. It was found that, rather than treating the stated institutional intention as a simple expression of older Chinese people’s likes or desires, the institutional care intention should be viewed as a compromise between their desire and the actual possibilities.

This study expanded the previous conceptual framework of the intention to live in institutions by integrating the Andersen model and the AFCIs. Our findings suggest that models should conceptualize residential and care intentions as a response to the interactions or expected interactions between older adults, their families, communities, and society as a whole. Policies and programs should build on each community’s capacity to develop a supportive environment for older residents to avoid insufficient institutionalization.

Regarding policy implications, our analyses indicate that the rapid growth of the institutional care sector may be misaligned with what the majority of older people need and want. Older adults are the best qualified to give a holistic perspective on their perceived care needs. Their voices should be considered in developing the aging service system (Zhou and Walker 2020). In addition, community development should be prioritized in building the aging service networks. Preventive health programs for older adults have more potential for promoting the health and well-being of Chinese older adults while being consistent with China’s economic and socio-cultural conditions. Meanwhile, the provision of institutional care should be based on an assessment of needs and avoid over-generalization. Further development of institutional care should emphasize the quality of care provided in institutions. A strengthened regulatory oversight eldercare institution is also required.

It should be noted that choice is central to the concept of later life arrangements and we also cannot ignore that some older adults gain better health and well-being from moving into an institutional setting [29]. Institutional care and aging in place are not necessarily a good option for everyone and will potentially cause harm if either is underdeveloped. To promote the health and well-being of older adults, policymakers and practitioners need to pay special attention to personal intentions and create affordable options based on these intentions.

## Figures and Tables

**Table 1:** Sample characteristics (*n* = 1937).

	All (*n*= 1937)	Institutional Care Intention (*n* = 278)	No Institutional Care Intention (*n* = 1659)	*p*
Predisposing factors				
Female	41.25	33.81	42.50	**
Age group				
60–64	22.93	26.28	22.37	
65–69	24.74	21.17	25.34	
70–74	19.33	18.25	19.52	
75–79	18.11	18.25	18.09	
80–84	10.96	12.77	10.66	
≥85	3.92	3.28	4.03	
Education				*
Below primary school	7.23	3.28	7.91	
Primary school	13.14	11.31	13.45	
Secondary school	24.68	24.82	24.66	
High school	28.19	32.12	27.52	
College or above	26.76	28.47	26.46	
Psycho-social factors				
Attitude towards public institutions				***
Poor	9.34	7.19	9.70	
Ok	22.72	27.34	21.94	
Good	17.81	27.34	16.21	
Not sure	50.13	38.13	52.14	
Attitude towards private institutions				***
Poor	11.41	13.31	11.09	
Ok	25.71	33.81	24.35	
Good	10.64	15.11	9.89	
Not sure	52.25	37.77	54.67	
Literacy about public institutions				
Don’t know	72.13	68.48	72.74	
Partially know	16.43	18.84	16.03	
Know	11.44	12.68	11.23	
Literacy about private institutions				***
Don’t know	76.51	64.75	78.48	
Partially know	14.61	22.30	13.32	
Know	8.88	12.95	8.20	
Enabling factors				
Married	76.08	75.09	76.25	
Household size				***
1	10.40	12.64	10.02	
2	41.77	46.93	40.89	
3	15.32	18.77	14.73	
4	11.34	11.19	11.37	
≥5	21.17	10.47	22.98	
Income				
A little short of money	13.19	15.50	12.79	
Successfully maintaining life	68.61	67.90	68.73	
Living a comfortable life	18.20	16.61	18.48	
Social support				
Yes	74.08	74.46	74.02	
Unknown	6.09	5.04	6.27	
Public transportation				
Satisfied	81.83	82.01	81.80	
Housing				
Satisfied	76.05	75.18	76.19	
Health service				
Satisfied	56.01	47.48	57.44	**
Community services				
Satisfied	64.33	57.55	65.46	*
Fitness and recreation facilities				
Satisfied	56.58	56.47	56.60	
Need factors				
Self-reported health				
Healthy	69.90	65.83	70.58	
Need for ADL assistance				
Yes	5.73	6.12	5.76	

Significant levels: * *p* < 0.05; ** *p* < 0.01; *** *p* < 0.001.

**Table 2:** Provincial characteristics.

Variables	All Sample		Institutional Care Intention		No Institutional Care Intention		*p*-Value
	Mean	SD	Mean	SD	Mean	SD	
Dependency ratio	12.25	3.05	12.47	3.02	12.21	3.06	
Institutional care beds per 1000 older persons	30.98	7.42	31.51	7.59	30.89	7.39	
Day care center beds per 1000 older adults	0.56	0.46	0.50	0.45	0.57	0.46	*
Activity centers for older adults per 1000 persons	1.61	0.88	1.63	0.80	1.61	0.90	
Beds of community health center per 1000 older adults	0.06	0.02	0.06	0.02	0.05	0.02	
*n*	1937		278		1695		

Notes: SD: standard deviation. Significant levels: * *p* < 0.05.

**Table 3:** Multilevel Logistic Analysis of Institutional Care Placement Intention (*n* = 1937).

Variables	Odds Ratio	SE	95% CI	
Gender (reference: female)	1.54 *	0.23	1.14	2.06
Age group (reference: 60–64)				
65–69	0.75	0.15	0.50	1.11
70–74	0.85	0.18	0.56	1.29
75–79	0.98	0.22	0.63	1.51
80–84	1.39	0.36	0.84	2.31
≥85	1.05	0.43	0.46	2.36
Education (reference: below primary school)				
Primary school	1.85	0.75	0.84	4.10
Secondary school	2.15 *	0.81	1.02	4.51
High school	2.33 *	0.87	1.12	4.85
College or above	2.19 *	0.83	1.04	4.61
Attitudes towards public institutions (reference: poor)				
Average	1.86 *	0.54	1.06	3.27
Good	2.88 ***	0.86	1.60	5.19
Not sure	1.37	0.39	0.78	2.40
Attitudes towards private institutions (reference: poor)				
Average	1.18	0.27	0.75	1.85
Good	1.09	0.30	0.64	1.88
Not sure	0.75	0.18	0.47	1.19
Literacy about public institutions (reference: don’t know)				
Partially Known	0.82	0.16	0.56	1.20
Know	0.66	0.16	0.41	1.06
Literacy about private institutions (reference: don’t know)				
Partially Known	1.56 *	0.30	1.07	2.28
Know	1.64 *	0.41	1.00	2.68
Marital status (reference: no spouse)				
Living with spouse	1.16	0.26	0.75	1.79
Household size (reference: 1)				
2	0.86	0.25	0.49	1.53
3	0.88	0.27	0.48	1.60
4	0.77	0.24	0.42	1.42
≥5	0.34 ***	0.11	0.18	0.66
Income (reference: short of money)				
Successfully maintaining life	0.84	0.18	0.56	1.27
Living a comfortable life	0.75	0.20	0.44	1.28
Social support (reference: no)				
Yes	0.96	0.17	0.68	1.36
Unknown	0.88	0.30	0.45	1.70
Public transportation (reference: dissatisfied)				
Satisfied	1.08	0.21	0.75	1.57
Housing (reference: dissatisfied)				
Satisfied	1.06	0.18	0.76	1.49
Health service (reference: dissatisfied)				
Satisfied	0.65 ***	0.10	0.48	0.87
Community services and environment (reference: dissatisfied)				
Satisfied	0.71 *	0.12	0.52	0.98
Fitness and recreation facilities (reference: dissatisfied)				
Satisfied	1.23	0.19	0.91	1.67
Self-reported health (reference: unhealthy)				
Healthy	0.82	0.13	0.61	1.12
Need for ADL assistance (reference: no)	1.05	0.32	0.58	1.90
Dependency ratio	0.96	0.03	0.91	1.02
Institutional care beds per 1000 older persons	1.02 *	0.01	1.00	1.05
Day care center beds Per 1000 older adults	0.56 **	0.12	0.37	0.85
Activity centers for older adults per 1000 persons	0.89	0.09	0.72	1.08
Beds of community health center per 1000 older adults	0.11	0.36	0.00	56.98

Notes: SE, standard error; CI, confident intervals. Significant levels: * *p* < 0.05; ** *p* < 0.01; *** *p* < 0.001.

## Data Availability

Restrictions apply to the availability of these data. Data was obtained from Research Centre for Medical Sociology and are available Jun Jing with the permission of Research Centre for Medical Sociology and Gallup China.
